# Femoral neck system versus inverted cannulated cancellous screw for the treatment of femoral neck fractures in adults: a preliminary comparative study

**DOI:** 10.1186/s13018-021-02659-0

**Published:** 2021-08-16

**Authors:** Yunfeng Tang, Zhen Zhang, Limin Wang, Wei Xiong, Qian Fang, Guanglin Wang

**Affiliations:** Department of Orthopaedics, West China Hospital, West China School of Medicine, Sichuan University, No. 37, Wuhou Guoxue Road, 610041 Chengdu, Sichuan People’s Republic of China

**Keywords:** Femoral neck system, Inverted cannulated cancellous screws, Femoral neck fracture

## Abstract

**Background:**

The femoral neck system (FNS) may be a valid alternative for treating femoral neck fractures, but few studies have compared the outcomes between FNS and cannulated cancellous screws. This study aimed to compare the clinical efficacy and complications of FNS with those of inverted cannulated cancellous screws (ICCS) for the treatment of femoral neck fractures in adults.

**Methods:**

This retrospective study evaluated patients with femoral neck fractures who underwent FNS or ICCS internal fixation between June 2019 and January 2020. The operative time, intraoperative blood loss, number of fluoroscopies, length of surgical incision, length of hospital stay, time to fracture healing, and other indicators were compared between the two groups. The incidence of complications such as nonunion, avascular necrosis of the femoral head, loosening of the internal fixation, and shortening of the femoral neck during follow-up was also assessed in both groups.

**Results:**

Among the 92 patients included, 47 and 45 patients were categorized into the FNS and ICCS groups, respectively. Follow-up ranged from 14 to 24 months. There were no significant between-group differences in sex, age, side of the injured limb, and type of fracture and in operative time, intraoperative blood loss, incision length, and length of hospital stay (all *P* > 0.05). The incidence of complications such as fracture nonunion, avascular necrosis of the femoral head, and screw loosening was also comparable between the two groups. However, the number of fluoroscopies was significantly lower in the FNS group than in the ICCS group (*P* < 0.05). The fracture healing time was also significantly lower in the FNS group than in the ICCS group (*P* < 0.05). Furthermore, the femoral neck shortening and Harris hip score at the last follow-up were significantly better in the FNS group than in the ICCS group (*P* < 0.05).

**Conclusions:**

FNS for femoral neck fractures improves hip functional recovery and reduces the femoral neck shortening rate and fluoroscopy exposure postoperatively. Thus, FNS can be an alternative to ICCS for the treatment of femoral neck fractures.

## Background

Considering the trends in population growth and ageing, the incidence of femoral neck fractures is projected to increase to 6.5 million by the year 2050, increasing the socio-economic burden and becoming a challenging problem for orthopaedic surgeons [1]. Femoral neck fractures in the elderly are mainly caused by low-energy trauma, such as a slight fall [2], while in young adults, they are commonly caused by high-energy trauma, such as traffic injuries [3, 4].

Orthopaedic surgeons face a significant challenge in treating femoral neck fractures because they need to take into account the following five independent variables: bone quality, fragment geometry, reduction, implant, and implant placement [5]. Roberts et al. reported that strong evidence supports arthroplasty for older patients with unstable (displaced) femoral neck fractures [6]. They also reported that moderate evidence supports operative fixation for older patients with stable (nondisplaced) femoral neck fractures [6]. In younger and more active patients with a femoral neck fracture, a number of studies have demonstrated that a satisfactory clinical outcome for the treatment of femoral neck fractures can be achieved by improving the reduction quality and getting a stable internal fixation [7–10]. Inverted cannulated cancellous screw (ICCS), dynamic hip screws (DHS) with or without anti-rotation screws, DHS with a blade instead of a screw, and similar implants are commonly used to treat such patients [11–14]. Thuan et al. and Protzman et al. have observed that the increased rates of internal fixation failure and nonunion were due to high shear forces and varus instability [4, 11]. Nonunion, femoral neck shortening, and screw-out are mostly complications associated with the internal fixation for treating femoral neck fractures [15–17]. The new minimally invasive implant femoral neck system (FNS), which was designed for dynamic stabilization of femoral neck fractures to reduce the above-mentioned complications, combines angular stability with a minimally invasive surgical approach [18]. However, few studies have compared the outcomes between FNS and ICCS in the treatment of femoral neck fractures.

The present study aimed to compare the short-term outcomes between the FNS group and ICCS group for femoral neck fractures. We hypothesized that the FNS group would yield superior outcomes compared to the ICCS group, with better perioperative status and improved postoperative function.

## Materials and methods

### Inclusion and exclusion criteria

After obtaining the Institutional Review Board approval, we performed a retrospective study, and informed consent was obtained from all including patients. The inclusion criteria were as follows: (1) the femoral neck system and inverted cannulated cancellous screw were used for surgical treatment respectively, (2) only unilateral fracture of the neck of femur, and (3) the follow-up time more than 12 months. The exclusion criteria included (1) pathological femoral neck fractures, (2) femoral neck fracture beyond 7 days, and (3) open fractures.

### Surgical procedures

**Fig. 1:**
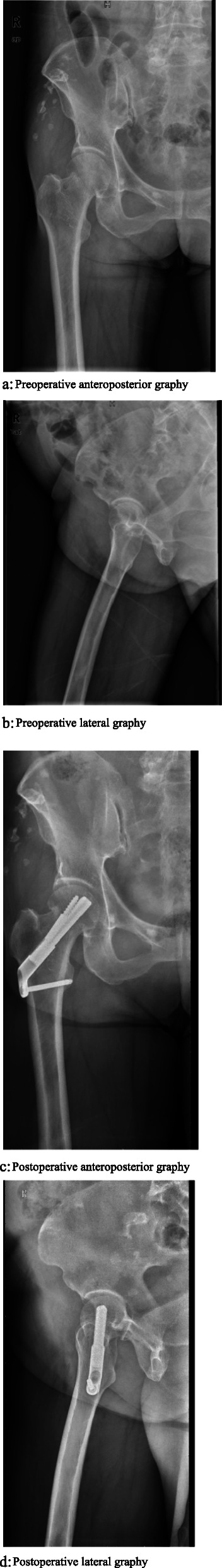
A 73-year-old female patient, right hip, operated at 18 h after trauma. **a** Preoperative anteroposterior graphy. **b** Preoperative lateral graphy. **c** Postoperative anteroposterior graphy. **d** Postoperative lateral graphy

**Fig. 2:**
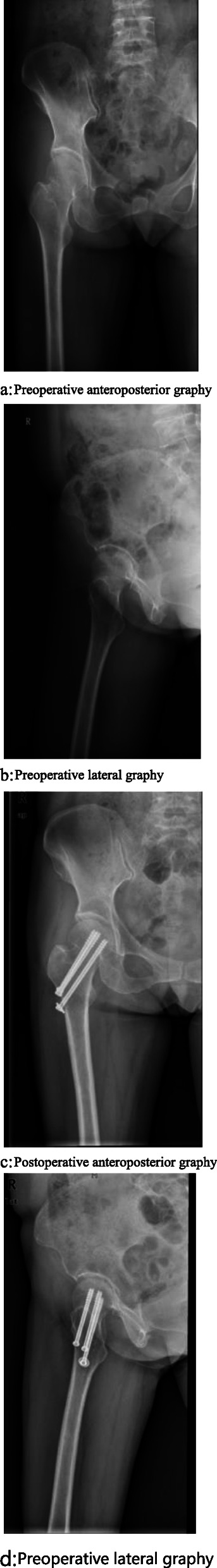
A 53-year-old female patient, right hip, operated at 12 h after trauma. **a** Preoperative anteroposterior graphy. **b** Preoperative lateral graphy. **c** Postoperative anteroposterior graphy. **d** Preoperative lateral graphy

After epidural or general anaesthesia, the patient was transferred to an orthopaedic traction bed. Open reduction was performed if the reduction did not meet the I and II levels under C-arm fluoroscopy, as assessed according to the Garden reduction index. A 4-cm incision parallel to the femoral shaft was made under the greater trochanter. In the ICCS group, three Kirschner wires forming an inverted triangle configuration, which were positioned approximately 5 mm beneath the surface of the femoral head cartilage, were inserted into the femoral head. After ensuring the proper location, 7.5-mm cannulated cancellous screws were inserted along the guide wires, and the Kirschner pins were removed. In the FNS group, a 2.5-mm guide pin was placed in the anterior superior part of the femoral neck to maintain fracture reduction. A 130° guide was inserted along the femoral neck. Under C-arm fluoroscopy, the needle insertion point and angle were adjusted by adjusting the guide so that the central guide was in the centre of the femoral neck in the anteroposterior and lateral positions, 5 mm from the subchondral bone. After reaming along the central guide, the bolt-and-plate assembly was inserted into the femoral head. Subsequently, the anti-rotation screw and locking screw were positioned in the proper location (Figs. 1 and 2).

### Perioperative management

Patients received second-generation cephalosporins 30 min preoperatively and 24 h postoperatively to prevent infection. Patients with stable fractures were allowed partial weight-bearing on the second day postoperatively. Meanwhile, patients with unstable fractures were allowed to undergo isometric contraction exercises of the quadriceps of the femoris and active and passive flexion and extension training of the ankle joint to reduce oedema of the lower extremities. Low-molecular-weight heparin (0.2–0.4 ml, according to the weight of patients) was routinely injected until discharge to prevent deep vein thrombosis.

Oral rivaroxaban and loxoprofen sodium tablets were prescribed at 10 mg/day and 180 mg/day (three times a day) for 3 weeks after discharge to prevent thrombosis and pain in a later stage. Partial weight-bearing and full weight-bearing exercises were gradually increased according to the rate of fracture healing on X-ray radiography. All patients were required to return to the outpatient clinic for follow-up at 1, 3, 6, 9, and 12 months postoperatively and then once every 6 months thereafter.

### Outcome measurement

All clinical data for operative time, incision size, surgical blood loss, length of stay, incidence of postoperative complications, Harris hip score (HHS), femoral neck shortening, and fracture healing time were assessed and recorded. Ischemic necrosis of the femoral head and nonunion were judged according to the criteria described by Dhar et al. and Slobogean et al. [19, 20]. The degree of shortening of the femoral neck was categorized as none/mild, < 5 mm; moderate, 5–10 mm; and severe, > 10 mm [21]. Hip function was assessed using the HHS published in 1969 which includes three sections of pain answered by patients, function, and range of motion assessed by the physiotherapist for deciding the patient hip function, with a total score of 100 [22].

### Statistical analysis

Continuous variables were expressed as mean ± standard deviation (SD) and were analysed by Student’s *t* test. The chi-square test was used to analyse categorical variables. Ranked data was tested by the Mann-Whitney *U* test. All statistical analyses were performed using SPSS 26.0 (SPSS, IBM, USA). *P* < 0.05 means the difference was statistically significant.

## Results

Finally, a total of 92 patients with femoral neck fractures were included in this retrospective study with following up 14–24 months. The FNS group and ICCS group, according to different internal fixation devices used, respectively included 47 patients and 45 patients. There are no statistically significant differences in terms of age, gender, injury sides, Garden classification, and Pauwels classification between the two groups (Table 1, all *P* > 0.05).

**Table 1:** Patient demographics between the FNS and ICCS groups

	FNS	ICCS	***P*** value
Cases	47	45	−
Gender (male/female)	34/13	37/8	0.259
Age (years)	57.4 ± 15.0	54.8 ± 11.7	0.44
Side (left/right)	26/21	23/22	0.686
Garden type			0.762
II	6	5	
III	29	31	
IV	12	9	
Pauwels type			0.884
I	5	6	
II	12	10	
III	30	29	

*Abbreviations*: *FNS* femoral neck system, *ICCS* inverted cannulated cancellous screw

No significant differences were found in terms of surgical time, intraoperative blood loss, incision size, and length of stay (*P* > 0.05). However, there was a statistically significant difference in fluoroscopy frequency (*P* < 0.05, Table 2).

**Table 2:** Comparison of surgical time, intraoperative blood loss, incision size, fluoroscopy frequency, and length of stay between the FNS group and the ICCS group

	FNS	ICCS	***P*** value
Fluoroscopy frequency (times)	7.6 ± 3.2	17.8 ± 3.8	<0.05
Surgical time (min)	52.4 ± 11.0	42.0 ± 11.9	0.590
Intraoperative blood loss (ml)	50.6 ± 10.6	47.3 ± 9.3	0.284
Incision size (cm)	4.0 ± 0.72	3.74 ± 0.59	0.214
Length of stay (days)	5.5 ± 1.3	4.8 ± 1.4	0.113

*Abbreviations*: *FNS* femoral neck system, *ICCS* inverted cannulated cancellous screw

In the FNS group, no or mild femoral neck shortness occurred in 34 (72%) of 47 patients, moderate shortening occurred in 11 (23%) patients, and severe shortening occurred in 2 (5%) patients. In the ICCS group, no or mild femoral neck shortness occurred in 25 of 45 (56%) patients, moderate shortening occurred in 9 (20%) patients, and severe shortening occurred in 11 (24%) patients. A significant difference was found between no/mild and severe shortening (*P* < 0.05). There was a significant difference in fracture healing time between the FNS group and the ICCS group (2.97 ± 0.35 months vs. 4.0 ± 0.39 months, *P* < 0.05). In the FNS group, nonunion occurred in 2 (4%) patients, osteonecrosis occurred in 1 (2%) patient, and screw-out occurred in 3 (6%) patients. In the ICCS group, nonunion occurred in 4 (9%) patients, osteonecrosis occurred in 3 (7%) patients, and screw-out occurred in 5 (11%) patients. At the last follow-up by outpatient or telephone, the mean Harris hip score of the FNS group was significantly higher than that of the ICCS group (88.9 ± 4.3 vs. 84.4 ± 3.2, *P* < 0.05) (Table 3).

**Table 3:** Comparison of postoperative follow-up between the FNS and ICCS groups

	FNS	ICCS	***P*** value
Fracture healing (months)	2.97 ± 0.35	4.0 ± 0.39	<0.001
Nonunion	2 (4%)	4 (9%)	0.430
Femoral neck necrosis	1 (2%)	3 (7%)	0.356
Screw-out	3 (6%)	5 (11%)	0.481
Femoral neck shortening			0.035
<5 mm	34 (72%)	25 (56%)	/
5–10mm	11 (23%)	9 (20%)	/
>10mm	2 (5%)	11 (24%)	/
Harris hip score	88.9 ± 4.3	84.4 ± 3.2	<0.05

*Abbreviations*: *FNS* femoral neck system, *ICCS* inverted cannulated cancellous screw

## Discussion

The increase in high-energy trauma, such as traffic injuries and high falls, has significantly increased the incidence rate of femoral neck fractures in young and middle-aged adults [23]. However, the majority of hip fracture patients are still the elderly, many of whom already have other major complications [24]. The outcomes of femoral neck fractures mainly depend on the physical status and age of the patient, type of fracture, quality of reduction, and stable internal fixation [25]. Implants for internal fixation of intracapsular femoral neck fractures can be divided into three categories: multiple cancellous screws, fixed-angle devices that allow sliding/compression, and fixed-angle devices that do not allow for sliding/compression [26]. Compared with fixed-angle fixation, multiple cancellous screws achieve better maintenance of bone stock, anti-rotation, and preservation of blood supply to the femoral head. However, the angle fixation device may have better resistance to varus deformity and micromotion than traditional inverted triangular screws [27, 28]. FNS combines the angular stability of sliding hip screws with the minimal invasiveness of multiple cannulated screws. Importantly, it shows good clinical results in resisting femoral neck shortening and complications.

Shortness of the femoral neck following internal fixation of femoral neck fractures is a known phenomenon, and clinicians who advocate for arthroplasty for the treatment of femoral neck fractures often use shortening as an argument against internal fixation of these fractures [29]. In our study, no/mild shortening in the ICCS group was present in 56% of the patients; moderate shortening, 20%; and severe shortening, 24%. Previous studies have reported a high incidence of shortening of the femoral neck after fixation with cannulated cancellous screws. Zlowodski et al. reported that the shortening rate was 31% for undisplaced fractures and 27% for displaced fractures [16]. Slobogean et al. reported that among patients younger than 55 years who underwent fixation with multiple cancellous screws, 22.8% and 12.9% developed moderate and severe shortening, respectively [30]. Consistent findings were observed in our study. There were significant differences in the rates of no/mild and severe shortening of the femoral neck between the ICCS and the FNS groups. In finite element analysis by Fan, FNS exhibited better von Mises stress distribution and smaller displacement of the femur than CCS in Pauwels III fracture at 50°, 60°, and 70° angles [31]. A biomechanical experiment by Stoffel proved that the loading test, starting at 500 N, increases at a rate of 0.1 N/cycle until the termination criteria are achieved. There was a significant difference of 15 mm in femoral neck shortening and leg shortening between the femoral neck system and cannulated cancellous screw (18171 ± 2585 N vs. 8039 ± 2778 N and 17372 ± 2996 vs. 7293 ± 2819, *P* < 0.001) [18]. Similarly, Schopper et al. concluded that FNS provides better resistance against varus deformity than Hansson pin systems for Pauwels II fractures (23007 ± 5496 N vs. 17289 ± 4686 B, *P* = 0.027) [16]. Collectively, the results of these biomechanical experiments and our outcomes indicate that the construct of FNS plays a key role in resisting femoral neck shortness. The lateral plate of the FNS makes the bolt, anti-rotation screw, and locking screw to form an angularly stable frame structure. On the one hand, similar to other angular stability constructs, the vertical shear stress can be distributed by the screw and lateral plate. On the other hand, the pre-collapsed insertion makes the anti-rotation screw and bolt slide in the maximum 20 mm packaging to meet femoral neck shortening during fracture healing.

Whether shortening of the femoral neck impairs hip function remains controversial. Zielinski observed that femoral neck shortening decreased gait velocity and impaired physical functioning in a multicentre clinical study of 75 patients [32]. Slobogean et al. found that among patients aged under 55 years, hip function at 12 months after fixation was lower in those with severe shortening of the femoral neck than in those with mild shortening [28]. Weil et al. reported a statistically negative correlation between the SF-12 score and overall neck shortening after fixation with multiple screws [33]. Haider also observed that among patients with femoral neck shortening, 92.5% of those with femoral shortening of ≥ 10 mm had a statistical trend toward a lower HHS. Nonetheless, the overall functional outcome was excellent, with a median HHS of 96 [34]. In the current study, the HHS was higher in the FNS group than in the ICCS group. Our data indicate that there might be an association between the extent of femoral neck shortening and the Harris score. The ICCS group had more severe shortness, which may be the reason for the significant difference in hip function between the FNS and ICCS groups.

Although femoral neck fracture reduction and fixation are performed in an attempt to restore vascular supply to the femoral head, 10–20% and 10–30% of patients develop complications of nonunion and avascular necrosis, respectively [35, 36]. In our study, the incidence rates of nonunion and femoral head necrosis were significantly lower in the FNS group than in the ICCS group. Cannulated cancellous screws can slide to the fractured interface and exert pressure on the fracture end, promoting bone union by increasing the stress stimulus to the fracture ends. Forming a three-dimensional frame with the femoral head is conducive to prevent the rotation of the femoral head, which in turn could avoid micro-displacement of the fracture ends. However, parallel inverted cannulated cancellous screws are distanced from each other, and it is difficult to constitute a standard triangle. Furthermore, in displaced fractures with a high risk of nonunion and ischemic necrosis, the biomechanical characteristics require rigid internal fixation to reduce the displacement of the fracture end after walking. Chen evaluated 44 patients with unstable and stable fractures who underwent fixation using a cannulated compression screw; within an average follow-up time of 27 months, 4.5% and 9.1% of the patients developed nonunion and avascular necrosis, respectively [37].

A clinical study by Ma et al. reported a 9.4% rate for both nonunion and osteonecrosis [38]. Considering their weak resistance to vertical shear and torsion, femoral neck fractures are being treated subsequently by fixed-angle devices, including dynamic hip screws and dynamic hip blades. These devices provide better angular stability and sliding compression; however, they also have limited efficacy in preventing anti-rotational stability, require longer incisions, and cause more soft tissue damage [39]. Furthermore, the hip screws and blade inserted into the femoral neck and head could compact the cancellous bone and destroy the surrounding blood supply. This may not be conducive to bone union and cause damage to the trabecular bone [40]. The FNS consists of one femur lateral plate with one or two holes for accommodating standard 5.0-mm locking screws, one bolt, and an anti-rotation screw. In fracture healing, statistical and dynamic compression play a key role after weight-bearing. After assembling the lateral plate and bolt, the unity forming a 130° angle structure is inserted into the femoral neck and femur shaft to achieve static compression and fixation of the fracture end. Concurrently, the bolt and the anti-rotation screw could slide along the lateral plate fixed by the locking screw to cause dynamic compression, acting like a dynamic hip screw. In addition, the anti-rotation screw prevents the rotation of the femoral head, reducing damage to new blood capillaries. Furthermore, recently published biomechanical evaluation and finite element analysis have supported that FNS provides better mechanical stability and is efficient in resisting vertical fractures [18, 31]. Therefore, we believe that the FNS might reduce nonunion and osteonecrosis by increasing stability, reducing damage to blood supply, and providing statistical and dynamic compression.

The design and mechanism of FNS for fixing femoral neck fractures are very similar to these of the Targon FN system, both of which provide an angular and rotator stable construct and offer a unique sliding mechanism, allowing for controlled fracture impaction [41]. However, the lateral incision over the greater trochanter of the femur was performed with a 5–10-cm incision length. Therefore, larger trauma with a larger amount of blood loss can significantly increase soft tissue exposure and may ultimately not be conducive to fracture healing [42]. Alshameeri et al. studied the treatment of neck fracture using the TFN over a period of 28 years [43]. They demonstrated a 2.7% nonunion rate after undisplaced fractures and 15.4% after displaced fractures. Takigawa et al. reported elective implant removal in 10.9% after undisplaced fractures and in 48.3% after displaced fractures, mainly due to discomfort around the implant [44]. In our study, there was a 4% rate of nonunion after surgery and no elective implant removal. We assume that FNS improves the ability of the sliding compressive mechanism and the size of FNS plate may be suitable for Asian people and reduces the irritation of the soft tissue around lateral plate. Compared with the results of the Targon FN, our results need further prospective evaluation of a multicentre, large sample randomized controlled clinical study.

We acknowledge some limitations of the current study. First, the retrospective study design might have resulted in a selection bias. Second, the relatively small sample size and short follow-up time might have an impact on clinical outcomes. Therefore, our results should be confirmed in larger multicentre randomized controlled trials with a longer follow-up period.

## Conclusion

FNS as a treatment for femoral neck fractures has comparable efficacy to ICCS with respect to operation time, incision length, surgical blood loss, and incidence of perioperative and postoperative healing complications. Moreover, FNS achieves superior Harris score, fracture healing time, and femoral neck shortening. Thus, FNS is a feasible alternative to ICCS for the treatment of femoral neck fractures.

## Data Availability

The datasets used and/or analysed during the current study are available from the corresponding author on reasonable request.

## Abbreviations

CCS: Cannulated cancellous screws
FNS: Femoral neck system
HHS: Harris hip score
ICCS: Inverted cannulated cancellous screws

## References

[1] Cooper C, Campion G, Melton LJ (1992). Hip fractures in the elderly: a world-wide projection. Osteoporos Int.

[2] Stevens JA, Rudd RA (2013). The impact of decreasing U.S. hip fracture rates on future hip fracture estimates. Osteoporos Int.

[3] Robinson CM, Court-Brown CM, McQueen MM, Christie J (1995). Hip fractures in adults younger than 50 years of age. Epidemiology and results. Clin Orthop Relat Res.

[4] Protzman RR, Burkhalter WE (1976). Femoral-neck fractures in young adults. The Journal of bone and joint surgery American volume.

[5] Kaufer H (1980). Mechanics of the treatment of hip injuries. Clin Orthop Relat Res.

[6] Roberts KC, Brox WT, Jevsevar DS, Sevarino K (2015). Management of hip fractures in the elderly. The Journal of the American Academy of Orthopaedic Surgeons.

[7] Szita J, Cserháti P, Bosch U, Manninger J, Bodzay T, Fekete K (2002). Intracapsular femoral neck fractures: the importance of early reduction and stable osteosynthesis. Injury.

[8] Krischak G, Beck A, Wachter N, Jakob R, Kinzl L, Suger G (2003). Relevance of primary reduction for the clinical outcome of femoral neck fractures treated with cancellous screws. Arch Orthop Trauma Surg.

[9] Heetveld MJ, Raaymakers EL, Luitse JS, Gouma DJ (2007). Rating of internal fixation and clinical outcome in displaced femoral neck fractures: a prospective multicenter study. Clin Orthop Relat Res.

[10] Estrada LS, Volgas DA, Stannard JP, Alonso JE (2002). Fixation failure in femoral neck fractures. Clinical Orthop Relat Res.

[11] Ly TV, Swiontkowski MF (2008). Treatment of femoral neck fractures in young adults. J Bone Joint Surg Am Vol.

[12] Gjertsen JE, Vinje T, Engesaeter LB, Lie SA, Havelin LI, Furnes O (2010). Internal screw fixation compared with bipolar hemiarthroplasty for treatment of displaced femoral neck fractures in elderly patients. J Bone Joint Surg Am Vol.

[13] Gurusamy K, Parker MJ, Rowlands TK (2005). The complications of displaced intracapsular fractures of the hip: the effect of screw positioning and angulation on fracture healing. J Bone Joint Surg Br Vol.

[14] Husby T, Alho A, Nordsletten L, Bugge W (1989). Early loss of fixation of femoral neck fractures. Comparison of three devices in 244 cases. Acta Orthop Scand.

[15] Karaeminogullari O, Demirors H, Atabek M, Tuncay C, Tandogan R, Ozalay M (2004). Avascular necrosis and nonunion after osteosynthesis of femoral neck fractures: effect of fracture displacement and time to surgery. Adv Ther.

[16] Zlowodzki M, Ayeni O, Petrisor BA, Bhandari M (2008). Femoral neck shortening after fracture fixation with multiple cancellous screws: incidence and effect on function. J Trauma.

[17] Wu Y, Leu TH, Chuang TY, Ho WP, Chen YP, Lin CY (2019). Screw trajectory affects screw cut-out risk after fixation for nondisplaced femoral neck fracture in elderly patients. J Orthop Surg (Hong Kong).

[18] Stoffel K, Zderic I, Gras F, Sommer C, Eberli U, Mueller D, Oswald M, Gueorguiev B (2017). Biomechanical evaluation of the femoral neck system in unstable Pauwels III femoral neck fractures: a comparison with the dynamic hip screw and cannulated screws. J Orthop Trauma.

[19] Dhar SA, Gani NU, Butt MF, Farooq M, Mir MR (2008). Delayed union of an operated fracture of the femoral neck. J Orthop Traumatol.

[20] Slobogean GP, Stockton DJ, Zeng B, Wang D, Ma BT, Pollak AN (2017). Femoral neck fractures in adults treated with internal fixation: a prospective multicenter Chinese cohort. J Am Acad Orthop Surg.

[21] Zlowodzki M, Brink O, Switzer J, Wingerter S, Woodall J, Petrisor BA (2008). The effect of shortening and varus collapse of the femoral neck on function after fixation of intracapsular fracture of the hip: a multi-centre cohort study. J Bone Joint Surg Br Vol.

[22] Harris WH (1969). Traumatic arthritis of the hip after dislocation and acetabular fractures: treatment by mold arthroplasty. An end-result study using a new method of result evaluation. J Bone Joint Surg Am Vol.

[23] Miyamoto RG, Kaplan KM, Levine BR, Egol KA, Zuckerman JD (2008). Surgical management of hip fractures: an evidence-based review of the literature. I: femoral neck fractures. J Am Acad Orthop Surg.

[24] Simunovic N, Devereaux PJ, Sprague S, Guyatt GH, Schemitsch E, Debeer J (2010). Effect of early surgery after hip fracture on mortality and complications: systematic review and meta-analysis. CMAJ.

[25] Smyth EH, Shah VM (1974). The significance of good reduction and fixation in displaced subcapital fractures of the femur. Injury.

[26] Hoshino CM, O'Toole RV (2015). Fixed angle devices versus multiple cancellous screws: what does the evidence tell us?. Injury.

[27] Swiontkowski MF, Harrington RM, Keller TS, Van Patten PK (1987). Torsion and bending analysis of internal fixation techniques for femoral neck fractures: the role of implant design and bone density. J Orthop Res.

[28] Linde F, Andersen E, Hvass I, Madsen F, Pallesen R (1986). Avascular femoral head necrosis following fracture fixation. Injury.

[29] Pauyo T, Drager J, Albers A, Harvey EJ (2014). Management of femoral neck fractures in the young patient: a critical analysis review. World J Orthop.

[30] Slobogean GP, Stockton DJ, Zeng BF, Wang D, Ma B, Pollak AN (2017). Femoral neck shortening in adult patients under the age of 55 years is associated with worse functional outcomes: analysis of the prospective multi-center study of hip fracture outcomes in China (SHOC). Injury.

[31] Fan Z, Huang Y, Su H, Jiang T. How to choose the suitable FNS specification in young patients with femoral neck fracture: a finite element analysis. Injury. 2021. 10.1016/j.injury.2021.05.043. 10.1016/j.injury.2021.05.043 34154816

[32] Zielinski SM, Keijsers NL, Praet SF, Heetveld MJ, Bhandari M, Wilssens JP, Patka P, van Lieshout E, FAITH Trial Investigators (2013). Femoral neck shortening after internal fixation of a femoral neck fracture. Orthopedics.

[33] Weil YA, Khoury A, Zuaiter I, Safran O, Liebergall M, Mosheiff R (2012). Femoral neck shortening and varus collapse after navigated fixation of intracapsular femoral neck fractures. J Orthop Trauma.

[34] Haider T, Schnabel J, Hochpöchler J, Wozasek GE (2018). Femoral shortening does not impair functional outcome after internal fixation of femoral neck fractures in non-geriatric patients. Arch Orthop Trauma Surg.

[35] Angelini M, McKee MD, Waddell JP, Haidukewych G, Schemitsch EH (2009). Salvage of failed hip fracture fixation. J Orthop Trauma.

[36] Haidukewych GJ, Rothwell WS, Jacofsky DJ, Torchia ME, Berry DJ (2004). Operative treatment of femoral neck fractures in patients between the ages of fifteen and fifty years. J Bone Joint Surg Am Vol.

[37] Chen C, Yu L, Tang X, Liu MZ, Sun LZ, Liu C, Zhang Z, Li CZ (2017). Dynamic hip system blade versus cannulated compression screw for the treatment of femoral neck fractures: a retrospective study. Acta Orthop Traumatol Turc.

[38] Dong Q, Han Z, Zhang YG, Sun X, Ma XL (2019). Comparison of transverse cancellous lag screw and ordinary cannulated screw fixations in treatment of vertical femoral neck fractures. Orthop Surg.

[39] Zderic I, Willhuber GC, Ahrend MD, Gras F, Barla J, Sancineto C, Windolf M, Richards G, Gueorguiev B (2019). Biomechanical comparison between standard and inclined screw orientation in dynamic hip screw side-plate fixation: the lift-off phenomenon. J Orthop Transl.

[40] Kold S, Bechtold JE, Mouzin O, Elmengaard B, Chen X, Søballe K (2005). Fixation of revision implants is improved by a surgical technique to crack the sclerotic bone rim. Clin Orthop Relat Res.

[41] Xiao YP, Shu DP, Bei MJ, Ji T, Kan WS, Li SG (2018). The clinical application of a novel method of internal fixation for femoral neck fractures-dynamic locking compression system. J Orthop Surg Res.

[42] Osarumwense D, Tissingh E, Wartenberg K, Aggarwal S, Ismail F, Orakwe S, Khan F (2015). The Targon FN system for the management of intracapsular neck of femur fractures: minimum 2-year experience and outcome in an independent hospital. Clin Orthop Surg.

[43] Alshameeri Z, Elbashir M, Parker MJ (2017). The outcome of intracapsular hip fracture fixation using the Targon Femoral Neck (TFN) locking plate system or cannulated cancellous screws: a comparative study involving 2004 patients. Injury.

[44] Takigawa N, Yasui K, Eshiro H, Moriuchi H, Abe M, Tsujinaka S, Kinoshita M (2016). Clinical results of surgical treatment for femoral neck fractures with the Targon(®) FN. Injury.

